# The Synergistic Effect of Combination Progesterone and Temozolomide on Human Glioblastoma Cells

**DOI:** 10.1371/journal.pone.0131441

**Published:** 2015-06-25

**Authors:** Fahim Atif, Neil R. Patel, Seema Yousuf, Donald G. Stein

**Affiliations:** Department of Emergency Medicine, Brain Research Laboratory, Emory University School of Medicine, Atlanta, GA, 30322, United States of America; Johns Hopkins University, UNITED STATES

## Abstract

Glioblastoma multiforme (GBM) is the most common and most aggressive malignant brain tumor. Despite optimal treatment and evolving standard of care, the median survival of patients diagnosed with GBM is only 12–15 months. In this study, we combined progesterone (PROG) and temozolomide (TMZ), a standard chemotherapeutic agent for human GBM, to test whether PROG enhances the antitumor effects of TMZ and reduces its side effects. Two WHO grade IV human GBM cells lines (U87MG and U118MG) and primary human dermal fibroblasts (HDFs) were repeatedly exposed to PROG and TMZ either alone or in combination for 3 and 6 days. Cell death was measured by MTT reduction assay. PROG and TMZ individually induced tumor cell death in a dose-dependent manner. PROG at high doses produced more cell death than TMZ alone. When combined, PROG enhanced the cell death-inducing effect of TMZ. In HDFs, PROG did not reduce viability even at the same high cytotoxic doses, but TMZ did so in a dose-dependent manner. In combination, PROG reduced TMZ toxicity in HDFs. PROG alone and in combination with TMZ suppressed the EGFR/PI3K/Akt/mTOR signaling pathway and MGMT expression in U87MG cells, thus suppressing cell proliferation. PROG and TMZ individually reduced cell migration in U87MG cells but did so more effectively in combination. PROG enhances the cytotoxic effects of TMZ in GBM cells and reduces its toxic side effects in healthy primary cells.

## Introduction

Human glioblastoma multiforme (GBM) is a highly proliferative brain tumor. The median survival of GBM patients remains only 12–15 months despite optimal treatment including surgical resection followed by radiation and Temozolomide (TMZ)-based chemotherapy [1]. Among the several limitations of current standard of care for GBM patients are incomplete tumor resection, peri-tumoral edema, blood-brain barrier (BBB) disruption, insufficiency of the maximum radiation dose to eradicate the tumor, the toxic side effects of chemo/radio therapy, and drug resistance.

TMZ, an oral DNA alkylating agent, is the current standard of care for the treatment of GBM and has been reported to increase survival by about 2 months when combined with surgery and radiation [1,2]. The mechanism of action of TMZ is based on its capacity to methylate DNA, which causes cellular cytotoxicity by forming O6-methylguanine adducts [2]. Unfortunately, GBM cells develop resistance to TMZ that is mediated by a DNA repair protein, O6-methylguanine-DNA-methyltransferase (MGMT), which removes TMZ-generated DNA adduct [3]. Resistance to TMZ is a major obstacle to treating GBM patients. It has been reported that GBM patients with a methylated MGMT promoter have increased overall survival and better response to combined TMZ and radiation therapy compared with radiation alone [4]. Lack of MGMT expression is considered a good prognostic factor in TMZ-treated GBM patients [5].

We propose that progesterone (PROG) in combination with TMZ might be effective in enhancing TMZ’s anti-proliferative effects while at the same time reducing some of its toxic side effects. PROG is a natural, neurosteroidal, developmental hormone synthesized in both males and females. It rapidly crosses the BBB and reduces inflammation and cerebral edema following traumatic brain injuries in pre-clinical and clinical studies [6,7]. In addition to its neuroprotective properties, PROG has been reported to exert anti-proliferative and apoptotic effects in breast, endometrial, ovarian, colon and salivary gland tumors *in vitro* and *in vivo* [8–11].

Examining the effects of PROG against human neuroblastoma and GBM in animals and cell culture models [12,13], we have found that high doses of PROG significantly decreased both neuroblastoma and GBM tumor growth but did not induce any cell death or significant proliferation in healthy and differentiated primary cortical neurons or human fibroblasts. PROG also enhanced the survival time of GBM tumor-bearing mice by ~60% [13]. Our investigation of possible mechanisms of action revealed that PROG inhibits tumor cell proliferation and angiogenesis and induces apoptosis in neuroblastoma and GBM tumors [12,13]. These findings strongly suggest that PROG over a specific range of doses, specifically can kill tumor cells without showing any demonstrable toxic side effects in healthy normal cells.

Here we hypothesize that PROG will enhance the anti-proliferative effects of TMZ and reduce some of its toxic side effects. It was logical to test this idea first in an *in vitro* model before testing in a mouse model because *in vitro* models are useful in screening novel drugs for safety and evidence of efficacy in relatively short periods of time. For our proof-of-concept research, we used an *in vitro* model of cytotoxicity to evaluate the anti-tumor effects of TMZ and PROG, alone or in combination, in two WHO grade IV human GBM cell lines, U87MG and U118MG. Both cell lines are derived from highly malignant GBM tumors and are highly invasive, as evidenced by their use in very recent cancer research articles [14–17]. We also tested whether PROG would reduce the toxicity of TMZ in healthy primary human dermal fibroblasts (HDFs). The sensitivity of GBM cells to TMZ treatment is known to be affected by many factors including the expression of MGMT and phosphorylation of Akt signaling [18]. Therefore, we examined the individual and combinatorial effects of PROG and TMZ on GBM cell proliferation and the EGFR/PI3k/Akt/mTOR signaling pathway, because this signaling is highly active in grade IV GBM. We also examined the effect of PROG and TMZ on the expression of MGMT in GBM cells as a marker of TMZ resistance. Finally, because GBM is highly infiltrative, we evaluated the effect of PROG alone and in combination with TMZ on the migration of U87MG cells.

The overall aim of this study was to test whether combined PROG will improve the efficacy of TMZ and reduce its toxic side effects in combination. Our *in vitro* data strongly suggest that PROG improves TMZ’s efficacy in GBM cells and reduces its toxicity in primary healthy cells.

## Materials and Methods

### Cell culture

WHO grade IV human GBM cell lines (U87MG and U118MG) were purchased from ATCC (Manassas, VA). PrimaPure HDFs were purchased from Genlantis (San Diego, CA). GBM cells lines and HDFs were cultured in Dulbecco’s Modified Eagle’s Medium containing 10% fetal bovine serum and fibroblast growth medium, respectively, at 37°C in a 5% CO_2_ environment according to manufacturer instructions.

### Cell death studies

U87MG, U118MG and HDF cells were seeded in a 24-well plate at a density of 0.5 x 10^5^/well. GBM cells were under starvation (serum-free condition) overnight prior to drug exposure. PROG (P3972, Sigma Chemicals, St. Louis, MO) and TMZ (34219; Sigma) stock was prepared in absolute dimethylsulfoxide (DMSO) and further diluted in culture medium. The final concentration of DMSO was kept at <5μl/ml. Cells were exposed to different concentrations of PROG (0.1, 1, 5, 10, 20, 40, 80 μM), TMZ (5, 10, 25, 50, 75, 100, 200 μM) and their combinations repeatedly for 3 and 6 days. For repeated exposure, culture medium was replaced daily and the drugs were added to the medium every day. Cell viability was measured at days 4 and 7. Concentrations for PROG and a repeated exposure protocol were selected from our previously published work, where we performed separate dose-response studies for PROG against human neuroblastoma and GBM tumors in nude mice after observing PROG’s beneficial effects at high doses in cell culture [12]. For TMZ, we selected the doses from previously published articles [19–22].

### MTT reduction assay

GBM cell viability was assessed by 3-(4,5-dimethylthiazol-2-yl)-2,5-diphenyltetrazolium bromide (MTT) assay as previously described [13]. The MTT assay is a standard, widely used method of measuring cell death/proliferation [23–26]. The reaction is based on the cleavage of the tetrazolium ring of the pale yellow MTT into dark blue formazan crystals by mitochondrial dehydrogenase enzyme in viable cells. Formazan crystals accumulate within the cells due to their impermeability to the cell membrane and are then solubilized by adding DMSO (50 ml). The intensity of blue formazan solution is directly proportional to the number of surviving cells. Concentrations were determined by photometric analysis. Briefly, 20 μl of MTT solution (5 mg/ml phosphate buffered saline (PBS)) was added per well and incubated at 37°C for 4 h until a purple precipitate was visible. DMSO (500 μl) was added to solubilize the crystals and the absorbance was read at 570 nm.

### Protein expression studies

Tumor cells (U87MG and U118MG) were seeded (0.5 x 10^6^) in 60-mm petri dishes and kept under starvation overnight prior to drug exposure. Cells were repeatedly exposed to different concentrations of PROG and/or TMZ (P5, P80, T100, P5+T100, P80+T100 μM) for 3 days. On day 4, cells were scraped from the petri dishes and the protein was extracted using an RIPA extraction buffer kit (Santa Cruz Biotechnology, Santa Cruz, CA) with protease inhibitors and assayed for protein concentration by bicinchoninic acid microplate protein assay (Pierce, 23225; Rockford, IL) [13]. Protein samples were prepared for Western blot analysis and analyzed for the expression of epidermal growth factor receptor (EGFR), total Akt, Phospho-Akt, mammalian target of rapamycin (mTOR), proliferating cell nuclear antigen (PCNA) and MGMT.

### Western blot

Protein samples (50 μg) were separated under reducing and denaturing conditions by 4–20% acrylamide Criterion gel (BioRad, Hercules, CA) at 200V for 1 h and transferred to a polyvinylidene difluoride membrane at 100V for 35 min. The non-specific binding sites of the membrane were blocked with 5% non-fat dry milk in PBS-T (PBS containing 0.05% Tween-20). The membranes were probed with the following primary antibodies overnight at 4°C: Akt (#9272); Phospho-AKT (#9271S); MGMT (#2739, Cell Signaling Technology, Danvers, MA); PCNA (#SC-56); EGFR (Santa Cruz #SC-03); mTOR (#32028, Abcam, Cambridge, MA); and β-Actin (AC74)lll (Sigma). Membranes were then incubated in their respective horseradish peroxidase-conjugated secondary antibodies. Blots were developed using a chemiluminescent substrate (Pierce) for 5 min. Chemiluminescent bands were detected on a Kodak autoradiography film in a darkroom and their densities were measured using NIH ImageJ software. In each figure, representative images were selected from the same gel.

### Cell migration assay

A monolayer scratch assay was performed as developed previously by Valster et al. [27]. We first examined the dose-response effect of PROG treatment on the migration of U87MG cells and then combined it with TMZ for combination treatment effects. In 12-well culture plates, 0.75 × 10^6^ cells were seeded per well. After the cells were attached and reached ~80% sub-confluence, they were incubated with starvation medium containing 2% fetal calf serum for 24 h prior to further incubation for 2 h in starvation medium in the absence (control) or presence of PROG (40 and 80 μM) or TMZ (50 and 100 μM) alone and in combination (P40+T50, P40+T100, P80+T50 and P80+T100). A scratch was then made through the cell monolayer with a sterile 200 μl pipet tip. Cells were washed with PBS, and photographs of the scratch area were taken in treated and untreated cells. For each well, two different areas of the scratch were photographed and their location on the dish was noted. Cells were further incubated with or without PROG for 24 h in starvation medium, the same areas were then re-photographed, and cells entering the denuded area were counted.

### Statistical Analysis

All data were expressed as mean ± standard deviation (SD). Statistical significance was set at *P*<0.05. Data were analyzed using one-way analysis of variance (ANOVA) followed by a LSD test. A two-tailed unpaired *t-*test was used to analyze Western blot densitometry. Analyses were calculated using SPSS 21.0 (IBM, Armonk, NY). Throughout this paper, “significant” can be understood to mean “statistically significant.”

## Results

### Effect of individual repeated treatment with TMZ and PROG on the viability of U87MG and U118MG cell lines

**Fig 1:**
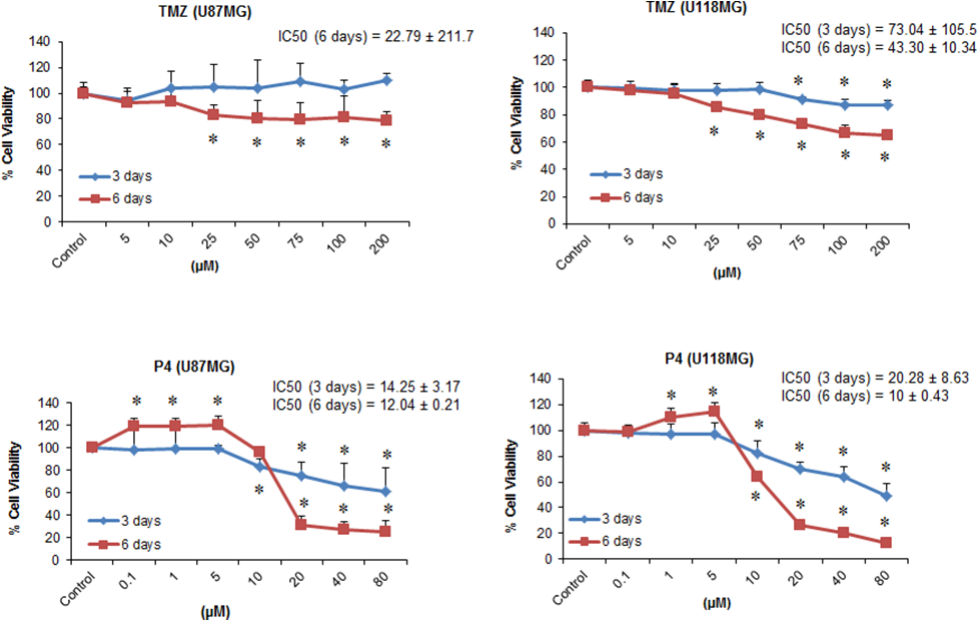
Effect of individual repeated treatment with TMZ and PROG on the viability of U87MG and U118MG cell lines. Cells were grown in 24-well plate and repeatedly treated with PROG and TMZ at different concentration for 3 and 6 days. For repeated exposure, culture medium was replaced daily and the drugs were added to the medium every day. On day 4 and 7, cell viability test was performed using MTT reduction assay. PROG and TMZ stocks were prepared in absolute DMSO and further diluted in culture medium. The final concentration of DMSO was kept at <5μl/ml. Data are expressed as means ± SD of three separate replication experiments (n = 3 each). Statistically significant difference: *P<0.05 compared with control group.

TMZ alone showed a significant group effect on the viability of U87MG cells following 6 days of exposure (F_(7, 40)_ = 2.49; *P*<0.03). Repeated exposure to different concentrations of TMZ alone for 3 days did not reduce the viability of U87MG cells even at the highest concentration (200 μM). After 6 days of repeated exposure, we observed a significant (*P*<0.05) decrease in cell viability at higher concentrations (25, 50, 75, 100, 200 μM), but the maximum decrease in cell viability was only ~20% (Fig 1). The half maximal inhibitory concentration (IC_50_) for TMZ was found to be 22.79±211.7 at 6 days in U87MG cells. In U118MG cells, we observed a dose-response effect of TMZ exposure on cell viability after 3 days (F_(7, 40)_ = 7.75; *P*<0.001) (IC_50_ = 73.04±105.5) and 6 days (F_(7, 40)_ = 49.25; *P*<0.001) (IC_50_ = 43.30±10.34) of repeated exposure (Fig 1). TMZ at the highest dose (200 μM) showed maximum cell death of 35% (*P*<0.05) compared to control following 6 days of exposure.

PROG alone produced significant cell death following 3 and 6 days exposure in both U87MG (F_(7, 40)_ = 6.0 and F_(7, 40)_ = 235.3, respectively; *P*<0.001) and U118MG (F_(7, 40)_ = 32.01 and F_(7, 40)_ = 557.95, respectively; *P*<0.001) cell lines in a concentration-dependent manner (Fig 1). Maximum cell death was observed at the 80 μM concentration following 6 days of exposure in U87MG (~74%) and U118MG (~87%) cell lines. In U87MG cells, the IC_50_ of PROG was found to be 14.25±3.17 and 12.04±0.21 following 3 and 6 days of exposure respectively. In U118MG cells, the IC_50_ of PROG was 20.28±8.63 and 10±0.43 following 3 and 6 days of exposure respectively.

### PROG enhances the anti-tumor effect of TMZ in GBM cell lines

**Fig 2:**
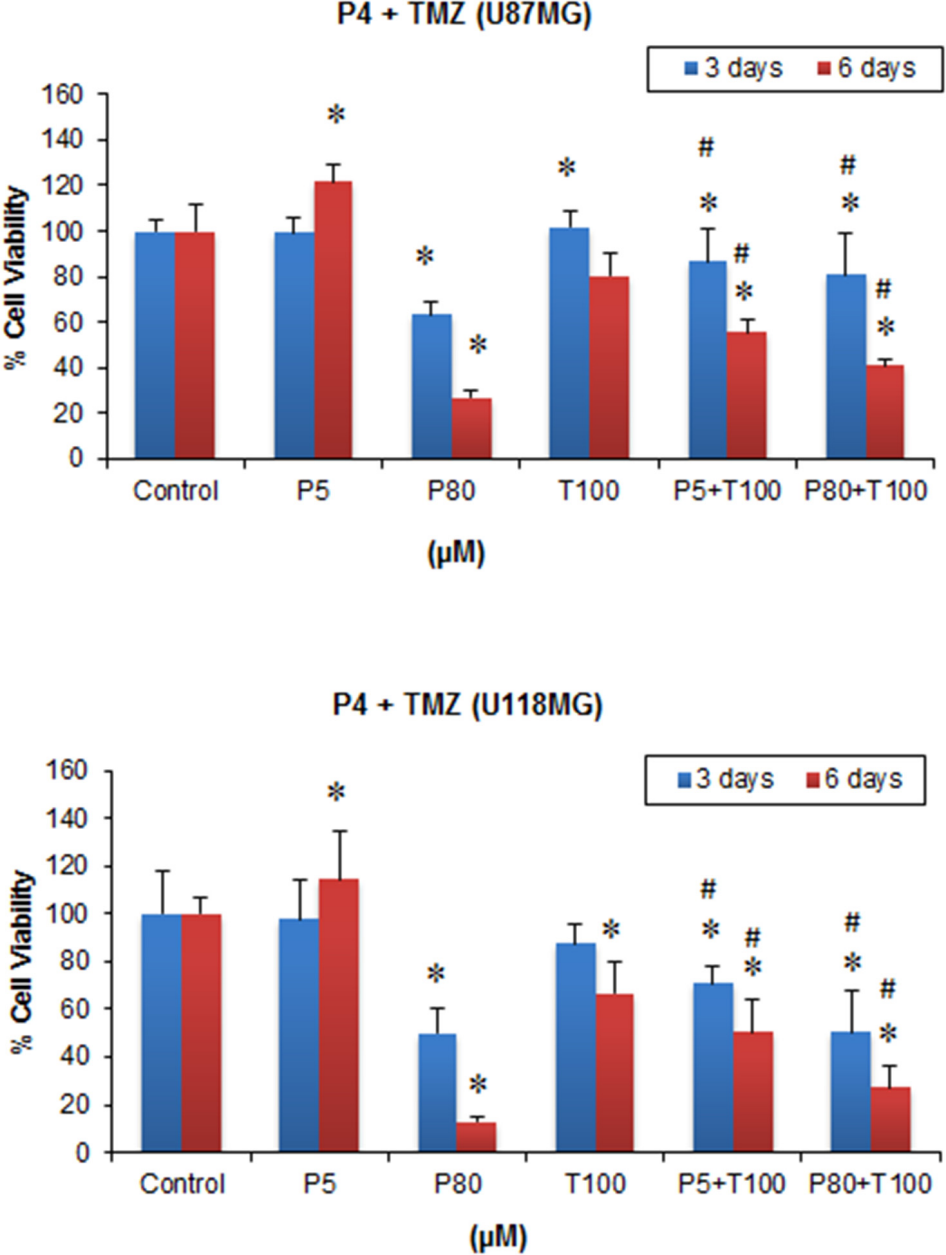
Effect of combined repeated treatment with PROG and TMZ on the viability of U87MG and U118MG cell lines. Cells were grown in 24-well plate and repeatedly treated with PROG and TMZ at different concentration for 3 and 6 days. For repeated exposure, culture medium was replaced daily and the drugs were added to the medium every day. On day 4 and 7, cell viability test was performed using MTT reduction assay. PROG and TMZ stocks were prepared in absolute DMSO and further diluted in culture medium. The final concentration of DMSO was kept at <5μl/ml. Data are expressed as means ± SD of three separate replication experiments (n = 3 each). Statistically significant difference: *P<0.05 compared with control group; ^#^ *P*<0.05 compared to T100 alone group. P5 = PROG (5 μM); P80 = PROG (80 μM); T100 = TMZ (100 μM).

When the GBM cells were treated with different combinations of PROG and TMZ, a significant group effect was observed following both 3 and 6 days’ exposure in both U87MG (F_(5, 42)_ = 14.6 and F_(5, 42)_ = 137.51, respectively; *P*<0.001) and U118MG cells (F_(5, 30)_ = 15.43 and F_(5, 30)_ = 63.27, respectively; *P*<0.001) (Fig 2). TMZ alone at 100 μM concentration, which was ineffective in reducing U87MG cell viability following 3 days’ exposure, produced a significant (*P*<0.05) reduction in viability when combined with PROG at 5 μM and 80 μM concentrations (~14% and 20%, respectively) after 3 days’ exposure compared to TMZ100 alone. This combination effect was more pronounced (*P*<0.05) after 6 days of exposure in P5 + TMZ100 and P80+ TMZ100 groups (30% and 49% respectively) compared to TMZ100 alone (Fig 2). In U118MG cells, P5+TMZ100 led to 19% and 24% more cell death (*P*<0.05) compared to TMZ100 alone after 3 and 6 days of treatment respectively. It is worth noting that P80+TMZ100 showed a significantly (*P*<0.05) better effect in reducing cell viability by 42% and 58% after 3 and 6 days treatment compared to TMZ100 alone (Fig 2).

### PROG reduces the toxicity of TMZ in HDFs

**Fig 3:**
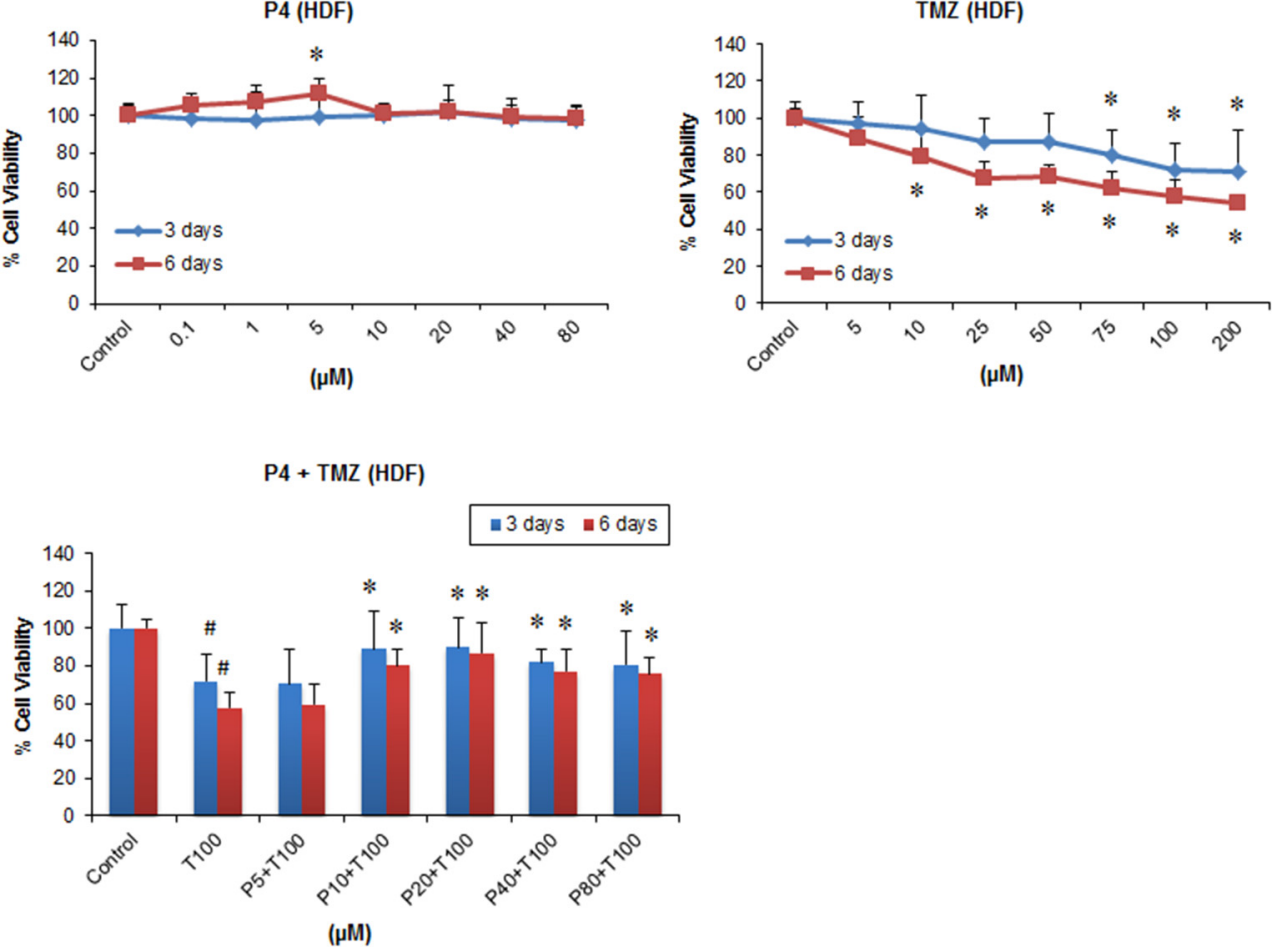
Individual and combined treatment effect of PROG and TMZ on the viability of primary human dermal fibroblasts (HDF). Cells were grown in 24-well plate and repeatedly treated with PROG and TMZ at different concentration for 3 and 6 days. For repeated exposure, culture medium was replaced daily and the drugs were added to the medium every day. On day 4 and 7, cell viability test was performed using MTT reduction assay. PROG and TMZ stocks were prepared in absolute DMSO and further diluted in culture medium. The final concentration of DMSO was kept at <5μl/ml. Data are expressed as means ± SD of three separate replication experiments (n = 3 each). Statistically significant difference: ^#^ *P*<0.05 compared to control group; *P<0.05 compared to T100 alone. P5 = PROG (5 μM); P10 = PROG (10 μM); P40 = PROG (40 μM); P80 = PROG (80 μM); T100 = TMZ (100 μM).

ANOVA showed no significant group effect on cell death following exposure to PROG alone for 3 days (F _(7, 40)_ = 0.094; *P*<0.998) and 6 days (F _(7, 40)_ = 2.11; *P*<0.065). *Post-hoc* tests revealed a significant (*P<*0.05) increase in HDF proliferation following PROG exposure at 5 μM concentration after 6-day exposures (Fig 3). In contrast, we observed a significant effect on the viability of HDF cells following TMZ exposure for 3 days (F_(7, 40)_ = 3.09; *P*<0.01) and 6 days (F_(7, 40)_ = 14.21; *P*<0.001). TMZ alone resulted in significant (*P<*0.05) cell death in HDF cells following 3 and 6 day exposures in a concentration-dependent manner (Fig 3). The maximum cell death was observed at 100 μM concentration following 3 days (~28%) and 6 days (~42%) of repeated exposure.

Next, we combined TMZ (100 μM) with different concentrations of PROG (5, 10, 20, 40, 80 μM) and examined their effects on HDFs. We observed a significant effect on the viability of HDF cells after 3 days (F_(6, 35)_ = 7.49; *P*<0.001) and 6 days (F_(6, 35)_ = 12.06; *P*<0.001) of combined exposure. PROG resulted in a significant (*P<*0.05) reduction in the cytotoxic effect of TMZ in healthy HDF cells following 3 and 6 days of treatment at all concentrations tested except 5 μM. PROG at 20 μM showed the maximum reduction in cell death following 3 and 6 days of combined exposure with TMZ (25% and 50% respectively) (Fig 3).

### Individual and combined treatment effect of PROG and TMZ on U87MG cell migration

**Fig 4:**
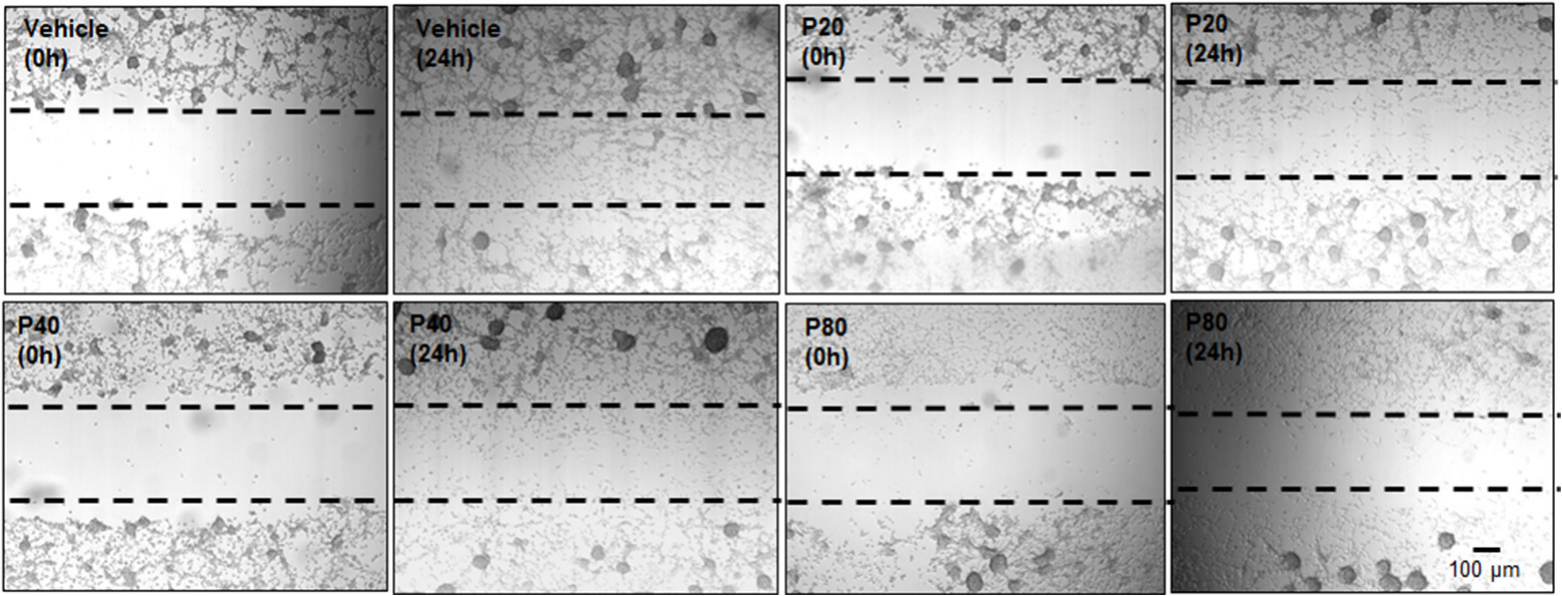
Individual treatment effect of PROG on U87MG cell migration in a wound healing assay. Cells were grown in multi-well plates and pre-treated with PROG alone at different concentrations for 2 h. A scratch/wound was formed with a 200-μl tip and the cells were incubated with PROG for the next 24 h. Photographs (4x) were taken at 0 h and 24 h post-wound formation. In the vehicle group, a large number of cells migrated from both sides to heal the wound at 24 h compared to 0 hr. PROG decreased U87MG cell migration 24 h after wound formation compared to vehicle. Representative photomicrographs from three separate replication experiments (n = 3 each).

We examined the effect of PROG alone (20, 40 and 80 μM) on cell migration of U87MG cells using qualitative wound-healing assay. We noted a marked increase in cell density in the scratch area in the vehicle group after 24 h of scratch formation (Fig 4). Compared to vehicle, PROG-treated cells showed a pronounced concentration-dependent decrease in cells migrating into the scratch area. PROG at 40 and 80 μM produced maximum inhibition. Our data suggest that at high doses PROG exerts anti-metastatic effects on GBM.

**Fig 5:**
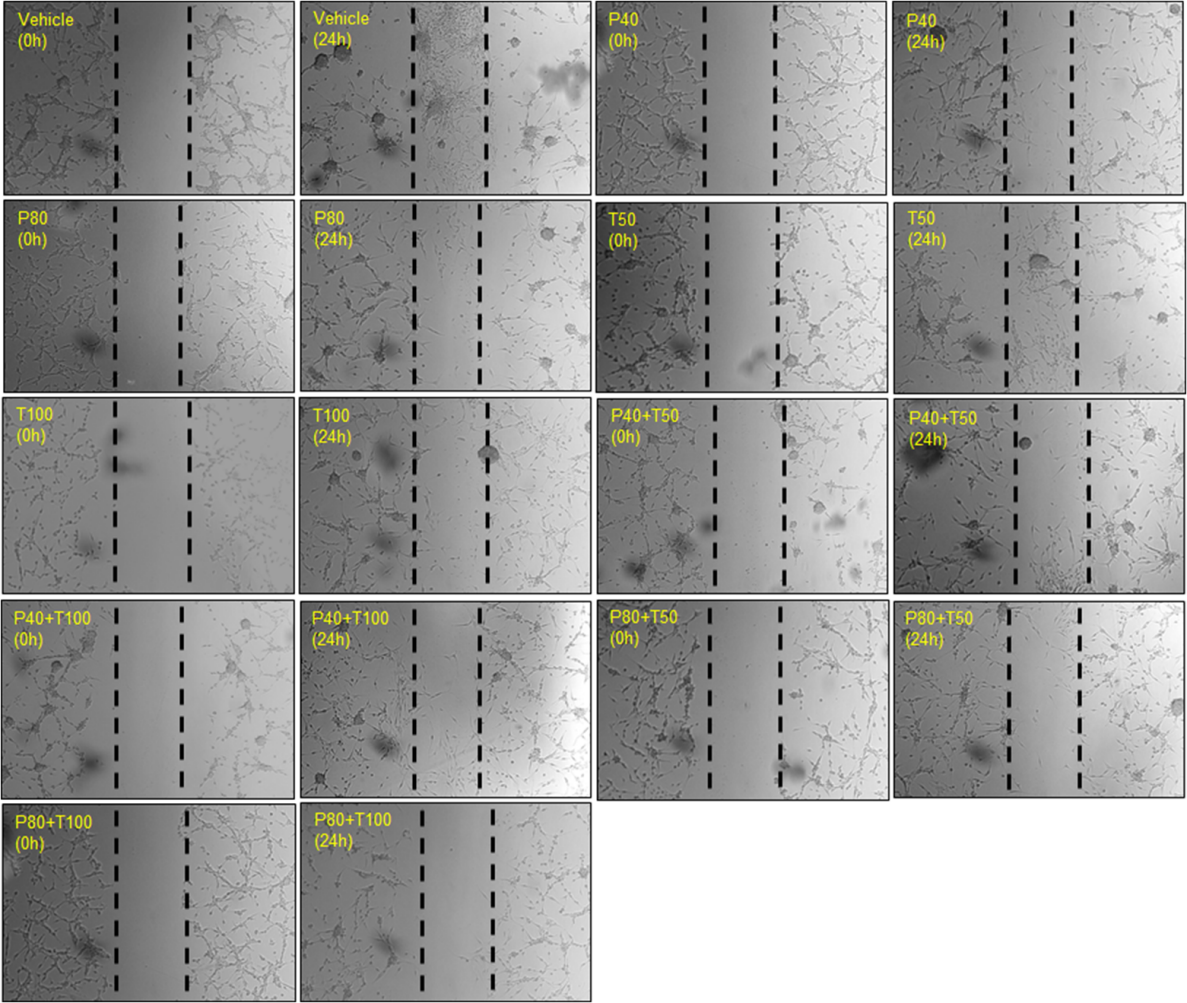
Combined treatment effect of PROG and TMZ on U87MG cell migration in a wound healing assay. Cells were grown in multi-well plates and pre-treated with **(A)** PROG alone and **(B)** in combination with TMZ at different concentrations for 2 h. A scratch/wound was formed with a 200-μl tip and the cells were incubated with PROG, TMZ or their combinations for the next 24 h. Photographs (4x) were taken at 0 h and 24 h post-wound formation. In the vehicle group, a large number of cells migrated from both sides to heal the wound at 24 h compared to 0 hr. PROG and TMZ individually decreased U87MG cell migration 24 h after wound formation compared to vehicle. The combination of the highest concentrations of both the drugs inhibited cell migration better than either drug alone. Representative photomicrographs from three separate replication experiments (n = 3 each).

TMZ alone produced a marked decrease in cell migration at all concentrations but was most effective at 100 μM (Fig 5). When we exposed U87MG cells to different combinations of PROG (40 and 80 μM) and TMZ (50 and 100 μM), we observed that the combined exposure was more effective than TMZ alone in inhibiting cell migration, and the most effective combination was P80+TMZ100. These data suggest that PROG enhances the anti-migration effect of TMZ in U87MG cells.

### Inhibition of EGFR/PI3K/Akt/mTOR signaling by combined PROG and TMZ exposure

**Fig 6:**
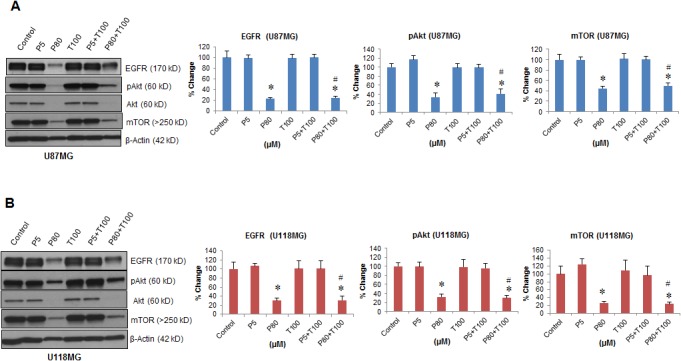
Effect of PROG and TMZ on the PI3k/Akt/mTOR signaling pathway in U87MG and U118MG cells. Tumor cells (U87MG and U118MG) were seeded (0.5 x 10^6^) in 60-mm petri dishes and kept under starvation overnight prior to drug exposure. Cells were repeatedly exposed to different concentrations of PROG and/or TMZ for 3 days. Protein samples (50 μg) were separated under reducing and denaturing conditions by 4–20% acrylamide Criterion gel and analyzed for EGFR, pAkt, total Akt and mTOR expression. The density of each P-Akt band was normalized with the density of corresponding total Akt band. β-actin was used as a loading control for densitometry. Representative Western blot and densitometric analysis of the expression of EGFR, phospho-Akt (Ser473) and mTOR in **(A)** U87MG and **(B)** U118MG cell lines. Data are expressed as means ± SD from two separate replication experiments (n = 3 samples each). Statistically significant difference: *P<0.05 compared to control; ^#^ *P*<0.05 compared to T100 alone.

Western blot data revealed a significant effect on the expression of EGFR in U87MG (F_(5, 30)_ = 146.31; *P*<0.001) and U118MG (F_(5, 30)_ = 27.38; *P*<0.001) cell lines (Fig 6A and 6B). A *post-hoc* test found a significant (*P*<0.05) decrease in EGFR expression in P80 and P80+TMZ100 compared to control which was significantly (*P*<0.05) better than TMZ100 alone in both cell lines. We observed a significant effect on the expression profile of both Akt and phosphorylated Akt (pAkt) in both U87MG (F_(5, 30)_ = 263.73; *P*<0.001 and F_(5, 30)_ = 84.91 respectively; *P*<0.001) and U118MG (F_(5, 30)_ = 27.82; *P*<0.001 and F_(5, 30)_ = 54.0 respectively; *P*<0.001) cell lines (Fig 6A and 6B). The maximum inhibition in Akt and pAkt expression was observed in P80 and P80+TMZ100, which were significantly (*P*<0.05) better than TMZ100 alone in both cell lines. Analysis of the mTOR expression profile revealed a significant group effect in U87MG (F_(5, 30)_ = 57.82; *P*<0.001) and U118MG (F_(5, 30)_ = 20.04; *P*<0.001) cell lines (Fig 6A and 6B). PROG alone and in combination with TMZ showed a significant reduction in mTOR expression in P80+TMZ100 compared to control and this inhibitory effect was significantly (*P*<0.05) better than TMZ100 alone in both cell lines. Individual and combined treatment with P5 and TMZ100 did not show any significant effect on the expression of EGFR, Akt, pAkt and mTOR expression in either U87MG or U118MG cell lines compared to controls.

### Synergistic effect of PROG and TMZ exposure on GBM cell proliferation

**Fig 7:**
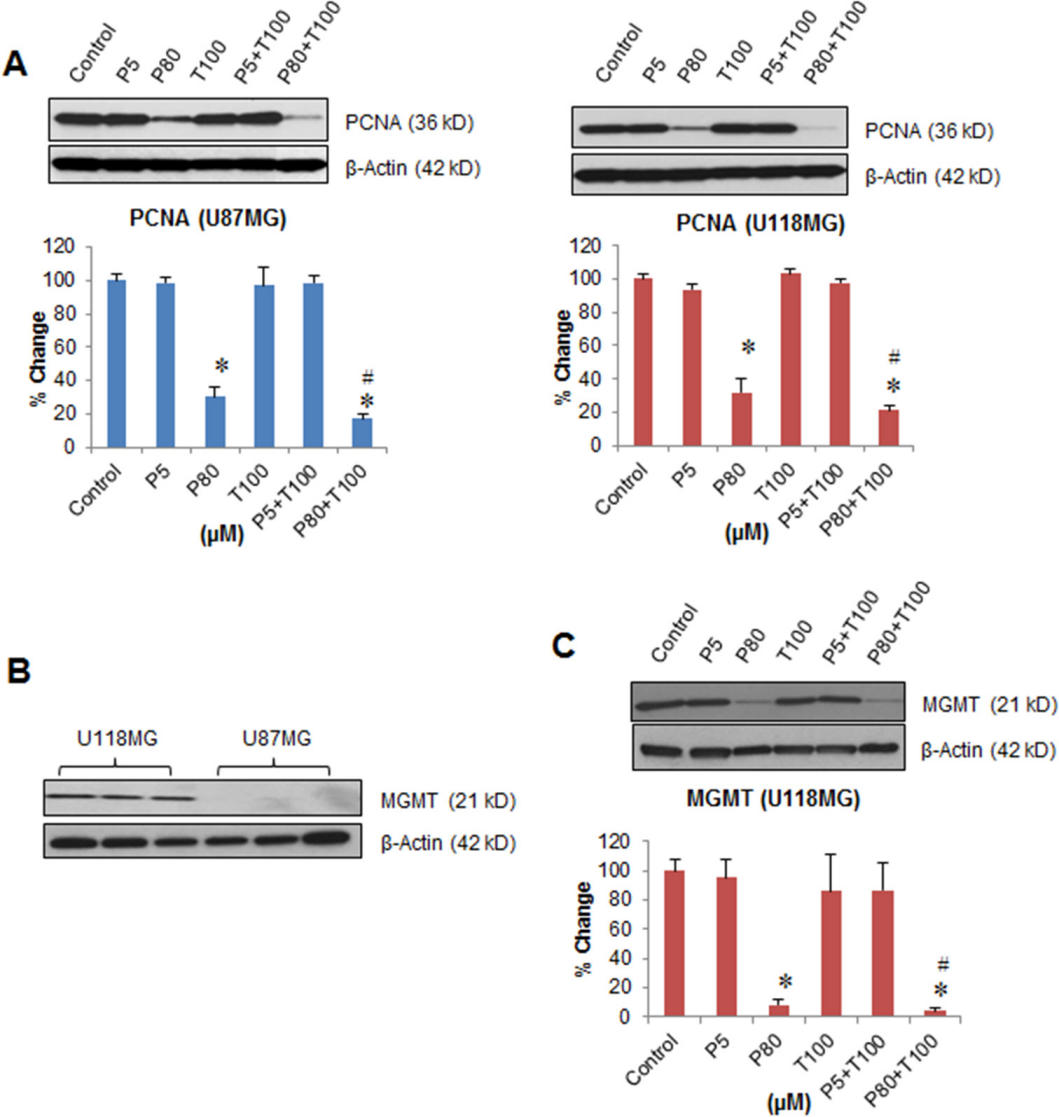
Effect of PROG and TMZ on the proliferation and the expression of MGMT in U87MG and U118MG cells. Tumor cells (U87MG and U118MG) were seeded (0.5 x 10^6^) in 60-mm petri dishes and kept under starvation overnight prior to drug exposure. Cells were repeatedly exposed to different concentrations of PROG and/or TMZ for 3 days. Protein samples (50 μg) were separated under reducing and denaturing conditions by 4–20% acrylamide Criterion gel and analyzed for EGFR, pAkt, total Akt and mTOR expression. The density of each P-Akt band was corrected for variance in loading, using the density of the corresponding total Akt. β-actin was used as a loading control for densitometry. **(A)** Representative Western blot and densitometric analysis of the expression of proliferation marker PCNA. **(B)** Expression of MGMT in U87MG and U118MG cells, and **(C)** Inhibitory effect of PROG on MGMT in U118MG cells. Data are expressed as means ± SD from two separate replication experiments (n = 3 samples each). Statistically significant difference: *P<0.05 compared to control; ^#^ *P*<0.05 compared to T100 alone.

We investigated the individual and combined effect of PROG (5 and 80 μM) and TMZ (100 μM, the best anti-tumor dose) exposures on the proliferation of U87MG and U118MG cells using the expression of PCNA as a marker of tumor cell proliferation (Fig 7A). A significant group effect on PCNA expression was observed in both U87MG (F_(5, 30)_ = 38.53; *P*<0.001) and U118MG (F_(5, 30)_ = 82.35; *P*<0.001) cell lines. A *post-hoc* test showed no significant difference in PCNA expression in either cell line after exposure to PROG (5 μM) and TMZ (100 μM) either alone or combination compared to controls. A significant (*P*<0.05) decrease in PCNA expression was observed in PROG (80 μM) and PROG (80 μM) + TMZ (100 μM) compared to controls and this was significantly (*P*<0.05) better than TMZ100 alone in both cell lines.

### Effect of PROG and TMZ on the expression of MGMT

First we determined the baseline expression of MGMT in U87MG and U118MG cells and found that it is highly expressed in U118MG but not in U87MG cells (Fig 7B). In U118MG cells, we examined the effect of PROG treatment on the expression of MGMT as a marker of TMZ resistance. We found a significant inhibitory effect of PROG (F_(5, 30)_ = 52.06; *P*<0.001) on MGMT expression (Fig 7C). At 5 μM concentration PROG alone did not show any effect on MGMT but at 80 μM it significantly (*P*<0.05) inhibited MGMT expression compared to the control group. TMZ (100 μM) alone did not show any effect on MGMT expression but combined with PROG (80 μM), there was a significant (*P*<0.05) inhibition in MGMT expression which was significantly better (*P*<0.05) than TMZ (100 μM) alone.

## Discussion

### PROG at high doses effectively inhibits the proliferation of grade IV human GBM U87MG and U118MG cells

Our data can be taken to demonstrate that PROG at high doses effectively inhibits the proliferation of grade IV human GBM U87MG and U118MG cells. TMZ alone also inhibits the rate of proliferation in these cells but not as effectively as PROG. When PROG was combined with TMZ, it reduced GBM cell viability better than TMZ alone. High-dose PROG alone reduced the migration of U87MG cells *in vitro*, suggesting a possible inhibitory effect of high levels of PROG on the infiltration of GBM tumor cells to the adjacent, still healthy brain tissue. TMZ also inhibited cell migration at high doses. The combined PROG and TMZ treatments produced a greater decrease in cell migration than either drug alone, suggesting that the combination therapy could help to contain the spread of the tumor *in vivo*. Our previous work shows that high-dose PROG reduces the toxic side effects of TMZ in primary human fibroblasts. Here, we observed an inhibitory effect of PROG and TMZ treatment on EGFR/PI3K/Akt/mTOR signaling, which is known to be highly active in GBM tumors and contributes to the high rate of abnormal cell proliferation [28].

### PROG exerts a dual hormetic effect

We interpret our cell death data to suggest that PROG exerts a dual hormetic effect in both U87MG and U118MG cell lines. PROG at low physiological concentrations enhanced tumor cell proliferation *in vitro* over 6 days of exposure, while at high, non-physiological concentrations, PROG inhibited proliferation. This dual effect could be explained by the fact that while PROG is a neurosteroid, it is also a natural growth hormone that plays a critical regulatory role in normal development [29]. In addition to affecting several normal physiological processes at low physiological concentrations, PROG levels become 9–10 times higher in pregnant compared to non-pregnant women [29], suggesting that PROG at high concentrations has a major role in regulating and controlling normal fetal growth. It is important to note that healthy fetal growth is exceptionally well controlled. PROG is one of many factors that inhibit abnormal growth by controlling the activity of positive regulators and inhibitors of the cell cycle [30]. Here we suggest that PROG exerts concentration-dependent hormetic effects which help to prevent abnormal/cancerous cell growth at high concentrations. Higher natural PROG levels during pregnancy are not only associated with a lower incidence of maternal breast cancer but also appear to exert a long-term protective effect against breast cancer [31]. Our findings are corroborated by recent studies in which we observed such dose-dependent, dual effects of PROG in several human GBM [13] and neuroblastoma [12] cell lines *in vitro*.

### TMZ alone is cytotoxic in U87MG and U118MG cells but is more effective in combination with PROG

Here we found that TMZ alone produced a cytotoxic effect in U87MG and U118MG cells following 3 and 6 days of repeated exposures. However, the maximum cell death observed was only ~20% in U87MG cells and ~35% in U118MG cells at high concentrations following 6 days of exposure. We noted a plateau effect of TMZ exposures at high concentrations. In brief, TMZ has been reported to induce cell death in different human GBM cell lines and our findings are in agreement with those observations [32–34].

Interestingly, when TMZ (100 μM) was combined with PROG at low (5 μM) and high (80 μM) concentrations, there was ~30% and 49% more cell death in U87MG cells compared to TMZ alone after 6 days of exposure. In the U118MG cells, the combination of TMZ and PROG at low and high concentrations produced ~24% and 58% more reduction respectively in cell viability compared to TMZ alone. These findings can be taken to indicate that PROG may enhance TMZ’s anti-tumor effects without producing side effects. The mechanisms underlying this synergistic effect remain to be explored. The combination of TMZ with PROG may act through different pathways at different concentrations. For example, we found that the cytotoxic effect of PROG at high concentrations in GBM and neuroblastoma cells is not mediated through the classical PROG receptor (PR) signaling pathway, while PROG’s proliferative effect at low concentrations is PR-dependent [12,13]. We speculate that the proliferative effects of low-dose PROG+TMZ in tumor cells makes them more chemosensitive for TMZ, resulting in more cell death.

A leading issue with current chemotherapeutic treatments is their non-specific cytotoxicity, which kills or maims not only the rapidly dividing tumor cells but healthy cells as well. An agent that does not affect the viability of healthy cells while it selectively kills cancer cells could potentially be an important advance for patient chemotherapy. We believe that PROG may be such an agent and worthy of further investigation. In the present study we repeatedly exposed primary human dermal fibroblasts to different concentrations of PROG for 3 and 6 days and observed that PROG application did not show any enhanced proliferative or cytotoxic effect, even at the same high concentrations that reduced the viability and migration of GBM cells. These data can be interpreted to suggest that, unlike other chemotherapeutic drugs, PROG at high doses may selectively kill tumor cells but apparently remains safe in healthy cells.

Next we examined the toxic side effects of TMZ at different concentrations in primary fibroblasts. Unlike PROG, TMZ killed the human fibroblasts in a concentration-dependent manner starting with doses as low as 10 μM. However, the maximum cell death observed was only 45%, induced at 200 μM following 6 days’ exposure. Our findings are supported by another paper suggesting that TMZ has less severe toxic effects compared to many other chemotherapeutic drugs [35]. When one of the most cytotoxic doses of TMZ (100 μM) was combined with different low and high doses of PROG (5, 10, 20, 40, 80 μM), we obtained a significant reduction in the cytotoxicity of fibroblasts at all combinations except P5+TMZ100 compared to TMZ alone. PROG at 10 and 20 μM concentrations showed the greatest reduction in fibroblast cell death in combination with TMZ. These findings are supported by our previous work showing that PROG at high concentrations (20, 40 and 80 μM) prevented excitotoxic cell death in primary cortical neurons after exposure to glutamate, and P20 afforded the greatest neuroprotection [36]. Taken together these findings support the idea that PROG has some degree of specificity in inducing the death of tumor cells while reducing the toxic side effects of TMZ in non-cancerous cells.

### Why is PROG alone better than its combination with TMZ in GBM cells?

Our data showed that PROG alone induced more cell death than TMZ alone or in combination. At this point we don’t know the exact mechanism underlying this finding. There may be several reasons for this. One of the strongest might be the fact that PROG is a pleiotropic hormone which exerts its beneficial effects by modulating a number of cell membrane/nuclear receptors, like non-classical membrane steroid receptors (mPRs, 25-DX and Sigma-1), a neurotransmitter receptor (GABA_A_), growth factor receptors (TrK family) and the classical nuclear steroid receptor (nPR). Upon activation, these receptors initiate their respective signaling pathways, which play critical roles in modulating excitotoxicity, proliferation, inflammation, apoptosis, trophic support, etc. Unlike TMZ, which acts mainly through DNA adduct formation, PROG therefore can act on different receptors and signaling pathways simultaneously to shut down the growth of GBM cells. When two drugs are combined, they may exert additive, synergistic, potentiating or inhibitory effects. We observed less cell death in the P80+T100 group compared to P80 alone. We speculate that this phenomenon may be the result of drug-drug interaction where TMZ blocked some of the PROG’s signaling pathways, contributing to GBM cell death. However, PROG enhanced the efficacy of TMZ in combination, which was the goal of this study.

Interestingly, PROG reduced the toxicity of TMZ in primary HDF cells even at high concentration (P80). Our data strongly suggest that P80 specifically kills tumor cells at high concentrations but remains safe in primary healthy cells at the same high concentrations. In contrast, chemotherapy drugs non-specifically kill every dividing cell. In combination, PROG is reducing the toxicity of TMZ through a mechanism/s which remains to be explored.

### PROG modulates both positive and negative regulators of the cell cycle

The cell cycle process is tightly controlled by several factors, including PROG. PROG modulates both positive and negative regulators of the cycle [30]. While we do not yet know how, we speculate that PROG can somehow differentiate between normal and cancer cells during cell cycling and compel tumor cells to undergo apoptosis, possibly by modulating the activity of the tumor suppressor gene p53. This idea is supported by our recent findings [13] showing that PROG induces cell death in p53 wild-type GBM cell lines (U87MG, U87dEGFR, U118MG) but not in p53 mutated cells (LN229). To confirm this hypothesis we will need to examine and compare the effects of PROG on different phases of the cell cycle in primary healthy cells and GBM cells.

### PROG inhibits the migration of U87MG cells in a concentration-dependent manner

Human GBM is highly invasive and infiltrates adjacent brain tissue, often without well-defined margins, making its surgical resection exceptionally difficult if not almost impossible [2]. Cell migration is a hallmark of GBM and one of the reasons it is so hard to treat [37]. In this study we examined the effect of PROG alone and in combination with TMZ on the migration of U87MG cells utilizing a wound-healing scratch assay. We found that PROG inhibits the migration of U87MG cells in a concentration-dependent manner. Next we combined PROG and TMZ and found that the cell migration into the scratch area was most effectively hindered by high concentrations of both PROG and TMZ (P80+T100). TMZ has been reported to modulate tumor cell migration [32,38], but to the best of our knowledge, we are reporting the first evidence for PROG’s anti-migration effect at high concentrations in U87MG cells.

### The role of the EGFR/PI3K/Akt/mTOR pathway in tumor proliferation

The therapeutic effect of TMZ is obstructed by at least two major factors. The first is the activation of the EGFR/PI3K/Akt/mTOR signaling pathway in GBM cells [18], a key signaling factor in the development of GBM mediating tumor proliferation [28]. EGFR amplification occurs in ~40% of primary GBM, with overexpression in over 60% of cases [39,40]. Mutation and overexpression of EGFR has been linked to the development of more aggressive malignant phenotypes leading to increased resistance to treatment and poorer clinical outcomes [41,42]. We looked at the EGFR/PI3K/Akt/mTOR pathway and found that PROG (80 μM) alone and in combination with TMZ (100 μM) inhibited the expression of EGFR in both U87MG and U118MG cell lines. TMZ alone did not show any effect on EGFR expression. Upon EGFR activation, PI3K is recruited to the cell membrane, which further activates Akt by phosphorylation at multiple sites. Phosphorylated Akt (pAkt) further activates multiple downstream targets, including mTOR, which are involved in cellular processes like metabolism, cell proliferation, cell growth and apoptosis [43,44].

### PROG (80 μM) alone and in combination with TMZ inhibits the expression of both pAkt (Ser437) and mTOR

Akt signaling has been reported to promote cell survival by activating the induction of cell survival proteins and blocking the function of pro-apoptotic proteins [44]. Our protein expression data revealed an inhibitory effect of PROG (80 μM) alone and in combination with TMZ on the expression of both pAkt (Ser437) and mTOR following 3 days of repeated exposure in both tumor cell lines. Again, TMZ alone did not have any effect on expression of these molecules. Because EGFR/PI3K/Akt/mTOR signaling promotes tumor cell proliferation, we next examined the effect of PROG and TMZ on the cell proliferation marker PCNA, which is often used for grading different neoplasms. PCNA is synthesized early in the G_1_ and S phases of the cell cycle as it forms a ring around DNA to facilitate and control DNA replication [45]. We found that P80 alone and in combination with TMZ100 significantly inhibited PCNA expression compared to control. Interestingly, this inhibitory effect was more pronounced, but not statistically significant, in combination than with PROG alone.

We also observed a significant decrease in cell viability in the P5+T100 group compared to the control group despite the fact that the expression of EGFR/pAkt/mTOR and PCNA remained unchanged. At this point we do not know the mechanism behind this observation but again, we speculate that the pleiotropic nature and non-genomic action of PROG are critical factors in such a response. PROG at low concentration acts through genomic pathways but at high concentrations through non-genomic pathways [12, 13]. Therefore it is very likely that PROG induces cell death in GBM cells not only through the EGFR/pAkt/mTOR pathway but also through other mechanisms which need to be explored in future work.

TMZ’s beneficial effect on tumor growth is impaired by drug-resistance, a major obstacle in the treatment of GBM [18]. The sensitivity of GBM cells to TMZ is inhibited by the expression of MGMT [3]. We observed that PROG at high, but not at low concentration, inhibits the expression of MGMT in U118MG cells following 3 days of repeated exposure. TMZ alone did not affect MGMT expression but led to a highly significant decrease in MGMT when combined with PROG at high concentration. Our data can be taken to indicate that PROG treatment inhibits the activation of both MGMT and EGFR/PI3K/Akt/mTOR signaling, which contributes to drug-resistance in GBM. Our findings suggest a possible role for PROG in reducing TMZ-resistance in GBM cells.

## Conclusions

PROG enhances the anti-tumor effects of TMZ and reduces its adverse side effects in non-tumor cells. PROG enhances the inhibitory effect of TMZ on the migration of GBM cells. The mechanisms underlying these effects clearly need further attention, but our data indicate a strong inhibitory effect of PROG alone and in combination with TMZ on the EGFR/PI3K/Akt/mTOR signaling which promotes tumor cell proliferation and inhibits apoptosis. The next step is to test our hypothesis in orthotopic animal models with implantation of human GBM neurosphere in the brain.
